# Evaluation of Stress Levels of Trainee Cardiac Surgery Residents during Training Interventions Using Physiological Stress Parameters

**DOI:** 10.3390/ijerph182211953

**Published:** 2021-11-14

**Authors:** George Awad, Robert Pohl, Sabine Darius, Beatrice Thielmann, Boris Kuzmin, Ingo Slottosch, Jens Wippermann, Hendrik Schmidt, Maximilian Philipp Scherner, Irina Böckelmann

**Affiliations:** 1 Department of Cardiothoracic Surgery, Otto-von-Guericke University Magdeburg, Leipziger Strasse 44, 39120 Magdeburg, Germany; george.awad@med.ovgu.de (G.A.); boris.kuzmin@med.ovgu.de (B.K.); ingo.slottosch@med.ovgu.de (I.S.); Herzchirurgie@med.ovgu.de (J.W.); maximilian.scherner@med.ovgu.de (M.P.S.); 2 Department of Occupational Medicine, Otto-von-Guericke University Magdeburg, Leipziger Strasse 44, 39120 Magdeburg, Germany; sabine.darius@med.ovgu.de (S.D.); beatrice.thielmann@med.ovgu.de (B.T.); irina.boeckelmann@med.ovgu.de (I.B.); 3 Clinic for Cardiology and Diabetology, Magdeburg Clinic, Birkenallee 34, 39130 Magdeburg, Germany; kardiologie@klinikum-magdeburg.de; 4 University Clinic for Internal Medicine III, Martin Luther University Halle-Wittenberg, Ernst-Grube-Straße 40, 06120 Halle, Germany

**Keywords:** heart rate variability, stress responses in surgeons, senior surgeons, surgery residents

## Abstract

Background: This study analysed the psychological and psycho-emotional stress in cardiac surgery. Using heart rate variability (HRV) analysis, it is possible to record intraoperative objective stress responses in surgeons. The aim of the study was to assess with the help of HRV parameters the postulated increased stress levels of cardiac surgeons in training compared to experienced senior cardiothoracic surgeons in exactly the same work situation in order to make qualification-differentiated statements about physiological stress during surgical interventions. Methods: During surgical teaching procedures, long-term ECG data (*n* = 15 each) for two operating residents and their assisting senior physicians were recorded. Time and frequency domain HRV parameters were analysed. Results: The time-related parasympathetic-dominated HRV parameters RMSSD (19.5 ms vs. 28.1 ms), NN50 (297.67 vs. 693.40), and cardiac interval mean RR (692.8 ms vs. 737.3 ms) indicate a higher stress level in the operating residents compared to the experienced surgeons. The higher stress index (11.61 vs. 8.86) confirms this. Conclusion: Compared to experienced surgeons, operating residents showed lower parasympathetic activity and higher stress levels during cardiac surgery training procedures.

## 1. Introduction

Compared to other occupational groups, the medical profession in general is associated with specific psychosocial stresses [1], and surgical physicians are exposed to additional risks and hazards. As various studies have shown [2,3,4], it is mainly work-related psychological stress that characterises the everyday life of a surgeon. The hazards resulting from work-related stress can have different health effects on those affected [5,6]. Burnout in particular has been a frequent component of diverse research related to stress identification in surgeons or physicians due to the presence of numerous long-lasting stressors that lead to impairment in the area of mental and psychosomatic health [7]. Prevalence estimates for burnout among physicians in Germany vary from about 4–20% [8]. A major part of the daily work in cardiac surgery consists of performing and assisting in surgical procedures. In this field of activity, surgeons are exposed to specific stressors, which can have an impact not only on the psychological strain, but also on intraoperative performance in the form of complications [9].

Training scenarios are crucial for the cardiac surgery profession, and there is limited availability of surgical simulators similar to those used in pilot training [10]. Moreover, these are not systematically used in training; most learning experiences occur during the operation itself. In this scenario, the senior surgeon also has a special position, especially due to the fact that he or she must constantly monitor and consider several processes (is the physician in training performing the current part of the operation adequately? What are the potential dangers that need to be pointed out? Is it responsible to let the junior surgeon also perform the upcoming steps, or is it necessary to take over the operation?). The above-mentioned processes are repeated continuously throughout the course of the operation, which also results in an especially tense situation for the instructor. In addition, the senior surgeon has the ultimate legal and medical responsibility for the course of the operation and for the patient.

Therefore, the aim of the current study was to determine:

1. The extent to which stress levels affect heart rate variability (HRV) in trainee cardiac surgeons.
2. The difference in stress levels between junior surgeon trainees and senior surgeons during cardiac surgery teaching procedures.
3. Whether preventive and health-promoting measures can be derived from the resulting findings and conclusions can be drawn for clinical practice, for example, in terms of arranging the operating room schedule.
4. Which valuable findings regarding stress should be generated for occupational groups in the context of occupational medicine and science.

However, mental stress in the operating room is difficult to measure. A number of vegetative and objective stress parameters to measure work stress/work strain have already been established [11]. Due to increasingly smaller measurement instruments and lower costs [12], the analysis of HRV is considered a feasible objective non-invasive measurement method in occupational medicine/occupational sciences in order to make statements about the degree of stress in a wide range of occupational groups as well as about the quality of the regulation of the cardiovascular system. In a review by Thielmann and Böckelmann 2016 [11], various studies related to the psychological stress of surgeons were pointed out, and it was confirmed that the heart rate increases in stressed surgeons and the HRV parameters show lower expressions.

## 2. Study Design and Sample Description

In a cooperative study by the Department of Occupational Medicine at the Otto-von-Guericke University Magdeburg and the Clinic for Cardiac and Thoracic Surgery of the University Hospital Magdeburg, the objective stress of residents and senior physicians during surgical teaching procedures in aortocoronary bypass (ACB) operations was investigated over a period of 6 months. The positive vote of the ethics committee of the Otto-von-Guericke University Magdeburg under the registration number 2020/185/19 is available, and the ethical standards of the Declaration of Helsinki in its currently valid version were observed. The current study is concerned with the analysis of recorded intraoperative stress parameters in assistant and senior physicians in the same work situation. On the basis of the results, statements will be made on the objective stress by means of the collected HRV parameters. Thus, the differences in stress levels during surgical teaching interventions between operating residents and assisting senior physicians will be shown.

The analysed ACB procedures were all performed using a heart-lung machine in mild hypothermia and cardioplegic arrest. These were elective procedures in patients with stable coronary 3-vessel disease. The ACB procedures considered for this study were performed in the daytime schedule starting in the second position of the surgical schedule, that is, usually between 11 am and 2 pm. This had the advantage that the influence of circadian rhythm on the HRV of the subjects could be ignored [13].

The physicians in training operated under the assistance of the senior physician volunteers. Intraoperative times were documented by an external person using a pre-prepared surgical protocol. In each case, the time from the start of the heart-lung machine to complete weaning from cardiopulmonary bypass was analysed.

The maximum number of surgical trainee residents within the clinic who could be considered and used as surgeons for teaching procedures is limited to two. This is a result of the number of appropriate training cases from across the surgical spectrum and the necessary frequency of training surgeries to achieve learning success. The two residents volunteered to participate in this collaborative study as subjects. The residents had previously performed 10 and 15 ACB procedures, respectively, under supervision. Both subjects had been in residency training for cardiac surgery for 6 years [14]. Before and during their surgical training they were able to participate in various training activities to learn surgical skills, for example, suture training in animal experiments or on cadavers. The attending senior physicians were also regularly involved in surgical training in the role of instructors.

## 3. Methodology, Heart Rate Variability Analysis, and Sample Description

The two male residents (age group 30–35 years) and the five senior physicians (age group 35–55 years) were healthy individuals without any pre-existing diseases. None of them were on any medications. Body mass index (BMI) breaks down within the sample between normal weight (1 resident with a BMI of 21.7 and 2 senior physicians with a BMI of 24.7 and 24.9) and slightly overweight (1 resident with a BMI of 29.7 and one senior physician with 27.4). The residents were non-smokers and there was one smoker among the senior physicians.

The data from the 30 24-h ECG recordings (2 ECGs each for 15 operations) of both groups of subjects served as the basis for the subsequent HRV analysis. The ECG recordings were made using a 2-lead ECG device (model MT-101, Schiller AG, Switzerland). The quality criteria of the Task Force of the European Society of Cardiology and the North American Society of Pacing and Electrophysiology [15] and of the AWMF-s2k guideline [12] were considered. The 24-h ECG recordings of all subjects were performed in sections of equal length and analysed in equal segments of the same length (180 min in the same work situation).

The data series from the NN intervals recorded at a sampling frequency of 1000 Hz was transferred to the Medilog DARWIN software. Artifacts were first automatically verified and in a second step, checked for plausibility by trained medical personnel; if necessary, these were manually corrected. The data series of successive cardiointervals was exported to a text file (.txt format) for subsequent HRV analysis and then processed using the Kubios HRV Premium program (Kubios, Kuopio, Finland) [16,17].

The mathematically calculated HRV parameters in the time and frequency domain as well as non-linear methods belong to the informative stress indicators. For the current study, selected HRV parameters were chosen (Table 1), which according to the AWMF-s2k guideline [12] allow conclusions to be drawn about mental stress that has occurred.

This HRV combines a variety of mathematically calculated parameters that characterize the variance, rhythmicity, or complexity of a time series of successive cardiac actions, the so-called NN intervals [12]. In this context, HRV is based to a significant extent on the tone of the vagus nerve, which excites the atria of the heart and modulates the self-sustained sinus rhythm of the sinus or Keith flack node. Especially in body rest and recovery phases, parasympathetic activity predominates and in a chronic stress state, sympathetic activity dominates [18]. This interplay of the sympathetic and parasympathetic nervous system can be estimated in a more differentiated way by HRV analysis during different demands [12].

The methods of HRV analysis or the HRV parameters obtained from them are divided into time, frequency, and nonlinear domains. In the analyses of the time domain, the NN intervals are mathematically evaluated with respect to their variance and reproduced in rhythm measures with a time dimension (usually ms) or in percentages. In the frequency domain, a power density spectrum is calculated from the stored RR series (NN intervals). In particular, the fast Fourier transform (FFT) and autoregression (AR) are the most widely used methods. In nonlinear analysis of HRV parameters, different methods are used to map the structure and complexity of heart rate time courses. These methods include the Lorenz plot or a trend-correcting fluctuation analysis [12].

In addition to the three main domain analyses, there is also the opportunity to gain an overview of the level of HRV parameters (divided into parasympathetic nervous system (PNS) tone and sympathetic nervous system (SNS) tone). In addition, it is possible to obtain a representation of the time periods in which subjects were in stress zones during a recording via the stress index [19].

### Statistical Analysis

IBM SPSS Statistics 26 (IBM Corp., Armonk, NY, USA) was used for statistical analysis. The Kolmogorov–Smirnov test was used to test the interval-scaled data for normal distribution. Homogeneity of variances was checked using Levene’s test. Depending on the equality of variances, the significance of the robust t test was determined. The level of significance was set to *p* < 0.05.

## 4. Results

Table 2 provides an overview of the time- and frequency-related HRV parameters used with the respective significance level (p). The mean values (mv) and the standard deviation (sd) of the HRV parameters of the residents were compared with those of the senior physicians.

All time-related parameters differ from each other (*p* < 0.05). The mean HR outside the operating room showed the same range in both groups (residents: 76.8 [1/min] vs. 77.7 [1/min] senior physician). Thus, both groups started with similar HR. Based on the lower parameters of mean RR (692.8 ms vs. 737.3 ms), RMSSD (19.5 ms vs. 28.1 ms), NN50 (297.67 vs. 693.40), as well as pNN50 (2.61% vs. 6.96%), the group of operating residents showed a higher stress level than the assisting senior physicians. This can also be seen in the higher mean HR value of 87.2 vs. 81.6 beats/min. The power density spectrum in the LF range (ms^2^) shows significantly higher values (*p* = 0.012) in the senior physicians (936.41 ms^2^ vs. 1658.90 ms^2^). The mean values of the LF/HF do not differ from each other (*p* > 0.05).

Table 3 contains the comparison of the stress HRV parameters of the examined groups.

All three of the listed parameters (Table 3) differ from each other in the two groups studied (*p* < 0.05). The activity of the parasympathetic part (parameter PNS-Index) was lower in the residents than in the senior physicians (−1.76 to −1.32). The activity of the sympathetic part, reflected in the SNS index, was higher in the residents (1.84) than in the senior physicians (1.01).

## 5. Discussion

Various studies have already used HRV to objectify stress in the medical profession. A systematic review on the workload of emergency physicians shows that the established parasympathetically mediated HRV parameters seem to be suitable parameters for objectifying stress [20]. Using HRV analysis, Mandegar and colleagues [21], for example, came to the conclusion that the type of surgery has an influence on the stress level of the surgeon. The stress level is higher in “off-pump” surgery (without heart-lung machine) than in “on-pump” coronary surgery (with heart-lung machine).

Demirtas [22] concluded, based on HRV analyses performed in surgical activities, that the assistance of a senior surgeon should be specifically included in studies related to psychological stress. The extent to which stress levels during surgical procedures are dependent on experience has hardly been investigated. While some studies make reference to this, there is potential for further surveys to elucidate differences between the stress levels of experienced and inexperienced physicians [23,24].

Performing operations in general is known to lead to a higher stress level [11]. In examining stress levels, our study focused on the comparison of residents in training and their senior facilitator. The collected physiological stress parameters of this study extend the existing research contributions on stress reactions in surgeons during surgical activities. Nevertheless, the results should not be generalized. On the one hand, the study presented here is a small sample, and on the other hand there is no control group or comparative HRV parameters from rest periods, for example. Moreover, in cardiac surgery, the use of a heart-lung machine and the ischemia time of the heart during coronary anastomoses are additional stressors due to the time factor, which is decisive in the course of the operation. Various factors that may have influenced the stress level of the surgeons could not be completely taken into account. These factors can lead to falsification of HRV values. These include workloads outside the operating room. The recording of sleep times and the consumption of beverages containing caffeine are based on the information provided by the subjects. Sleep-related impairments may be associated with clinical treatment failures in physicians [25]. Whether caffeine consumption can influence cardiovascular autonomic activity has not yet been adequately evaluated [26]. Nevertheless, caffeine should be avoided for half an hour before the measurement [12]. Within the study, there may be possible bias due to age as a confounding factor. The influence of age on HRV [27] and the occurring decline in cognitive and physical abilities should be considered in similar HRV analyses. One further aspect is that one of the analysed subjects is a smoker. Smoking is not a contraindication for HRV measurements [12]. Active smoking can affect heart rate and lead to a reduction in HRV [28,29]. However, as our proband was not a heavy smoker and smoking normally took place in the evening hours, we did not consider this as a significant influence on our HRV measurements.

The higher sympathetic activities of the residents confirm that young surgeons are exposed to a particularly high level of stress in the operating room. This is especially evident in our data when looking at the stress index and the defined stress zones of Tarvainen et al. [19] following the Baevski index. There, both values of the physician groups were in the normal range (7.1–12.2), but the residents nevertheless showed a higher sympathetic activity (11.61) than the senior physicians (8.68). In the RMSSD zones defined according to Tarvainen et al. [19], the assistant physicians with an RMSSD of 19.5 ms are in the lower zone (12–27 ms) and the senior physicians with 28.1 ms are in the normal zone (27–72 ms). In this respect, in comparison to the senior physicians, lower parasympathetic activity can be seen in the residents. During the survey, the senior physicians participating in the study were exposed to the challenge of providing adequate guidance to their younger colleagues, and at the same time bearing the responsibility for a complication-free surgical procedure. Nevertheless, the HRV parameters among assisting senior physicians showed less stress and more relaxation during coronary surgery teaching procedures.

Rieger et al. [23] analysed the intra-individual workload differences between surgeon and assistant using the objective physiological parameters of heart rate, respiratory rate, and skin temperature. Unlike the current study, in Rieger et al., the work of a primary surgeon did not lead to a higher workload; however, the setting of surgical training was not investigated. When analysing the stress levels of experienced and inexperienced surgeons during a cardiac surgery procedure, Kuhn et al. [24] concluded that surgical experience was not associated with reduced stress levels. However, this work was based on analysis of the heart rate and sympatho-vagal balance. In summary, the current work represents the first comprehensive study of stress within cardiac surgery training based on detailed statistical analysis of the HRV parameters shown.

Prichard et al. [30] studied HRV parameters in experienced and less experienced surgeons in a cross-over design with 50 thyroidectomies and 50 lobectomies each. There, the more inexperienced surgeons showed a higher stress level when operating than when assisting. Despite a lack of statistical significance, this was shown by lower RMSSD and pNN50 parameters as well as by a higher LF/HF ratio. This is in agreement with the results of the current study, where the parameters used to detect parasympathetic activity (RMSSD, NN50, and pNN50) also have lower expressions. For the remaining parameters from the time domain, both sympathetic and parasympathetic activity are discussed [18].

Taking into account the fact that many stressors in the daily work of residents also occur outside the operating room (including ward work, night and emergency duties, the degree of responsibility towards patients and superiors [31], and that the medical profession is characterized by above-average working hours), young physicians belong to a group that is at high risk of health problems.

Reference values and analysis possibilities of HRV were already mentioned in 1996 in the recommendations of the Task Force of the European Society of Cardiology and the North American Society of Pacing and Electrophysiology [15]. The results presented here can only be compared with these to a limited extent. The reference values published at that time were based on HRV analyses lying down and on 5 min time intervals. In addition, studies show that significant differences in the analysed HRV parameters exist when directly comparing old tape recorder systems (from the recommendations of the European Society of Cardiology and the North American Society of Pacing and Electrophysiology) vs. the newer hard disk recorders [32].

The professional group of physicians and especially the specialist discipline of surgeons should be the focus for early preventive approaches with regard to psychological stress in the future. Young physicians in particular should be considered as a potential target group for interventions and, if necessary, therapeutic measures. Resilience training can be used to learn methods and techniques to strengthen one’s own resilience. Studies on the benefits of resilience programs among healthcare professionals (including physicians) suggest at least short-term benefits. After resilience training, they show a short-term increase in resilience and decrease in depression symptoms [33]. Prospective surgeons might benefit from structured training to increase their perceived stress resources or stress coping skills.

It must be taken into account that the acquisition of surgical skills creates high levels of stress for young surgeons. Nevertheless, early skill acquisition as well as stress adaptation strategies should be encouraged with the help of regular training programs (e.g., Skills Lab). It has been shown that (in laparoscopic procedures) there is indeed a correlation between skill improvements and stress reduction in novice surgeons. There is a correlation (in laparoscopic procedures) between skill improvement and stress reduction in trainee surgeons [34]. This indicates that repeated confrontation with a learning task can promote positive stress adaptations. The relationship between skill improvement and stress reduction in trainee surgeons has also been demonstrated [34]. Studies on the effects of stress management courses among physicians are scarce. However, surveys of students do show positive effects on physiological stress parameters [35,36]. There is still a need for research using HRV analysis to demonstrate the effects of different training programs on the stress levels of surgeons.

## 6. Conclusions

The present study analysed physiological stress responses during cardiac surgery training procedures using HRV analysis. Our study indicates that less experienced residents exhibit higher psychological stress levels than the more experienced supervising senior surgeons. Therefore, focus on well-designed training programs might lead to improved stress management and an enhanced learning progress. Further evaluation of stress response during cardiac surgery procedures is required.

## Figures and Tables

**Table 1:** Explanation of the HRV parameters used according to the AWMF-s2k guideline [12].

HRV Parameters	Explanation	Activity as Part of the Autonomic Nervous System
Time domain parameters		
Mean HR [1/min]	Heart rate	Sympathetic and parasympathetic nervous system
Mean RR or NN [ms]	Distance between two R-pins or NN-intervals	No clear assignment
RMSSD [ms] (Root Mean Square of successive differences)	Square root of the mean of the sum of all squared differences between adjacent RR intervals	Parasympathetic nervous system
NN50	Number of consecutive RR intervals that differ from each other by more than 50 ms	Parasympathetic nervous system
Frequency domain parameters		
FFT_LF_po [ms^2^]	Power density spectrum in the frequency range from 0.04 to 0.15 Hz	Sympathetic and parasympathetic nervous system
FFT_HF_po [ms^2^]	Power density spectrum in the frequency range from 0.15 to 0.40 Hz	Parasympathetic nervous system
FFT LF/HF	Measure of sympatho-vagal balance as quotient of LF and HF	Sympathetic and parasympathetic nervous system
Stress, PNS, and SNS Index		
Stress Index	The stress index is the square root (to make the index normally distributed) of the Baevsky stress index, where values from 150–500 are claimed as the normal range (12.2–22.4 when the square root is applied)	Sympathetic nervous system
PNS index	Activity of the parasympathetic nervous system described by the mean values of RR, RMSSD, and SD 1(%)	Parasympathetic nervous system
SNS index	Sympathetic nervous system activity described by mean values of mean HR, stress index, and SD2 (%)	Sympathetic nervous system

**Table 2:** Comparison of selected time-related HRV parameters as well as parameters from FFT analysis during ACB interventions in the cannulation to decannulation phase between the operating residents (*n* = 15) and the assisting senior physicians (*n* = 15).

Parameter	Residents			Senior Physicians			*p*-Value
	mv		sd	mv		sd	
Mean RR [ms]	692.8	±	56.59	737.3	±	42.43	0.021
Mean HR [1/min]	87.2	±	7.34	81.6	±	4.85	0.022
RMSSD [ms]	19.5	±	6.42	28.1	±	9.69	0.008
NN50	297.67	±	379.03	693.40	±	479.68	0.018
FFT_LF_po [ms^2^]	936.41	±	491.49	1658.90	±	893.40	0.012
FFT_HF_po [ms^2^]	148.05	±	105.99	276.42	±	266.82	0.094
FFT_LF/HF	7.2	±	1.76	7.2	±	2.84	0.974

**Table 3:** Comparison of the stress HRV parameters during ACB interventions in the cannulation to decannulation phase between the operating residents (*n* = 15) and the assisting senior physicians (*n* = 15).

Parameter	Residents			Senior Physicians			*p*-Value
	mv		sd	mv		sd	
PNS Index	−1.76	±	0.32	−1.32	±	0.45	0.004
SNS Index	1.84	±	0.62	1.01	±	0.56	0.001
Stress Index	11.61	±	2.17	8.68	±	1.61	0.000

## Data Availability

The data can be requested from the author team.
